# *Desulfotomaculum* spp. and related gram-positive sulfate-reducing bacteria in deep subsurface environments

**DOI:** 10.3389/fmicb.2013.00362

**Published:** 2013-12-02

**Authors:** Thomas Aüllo, Anthony Ranchou-Peyruse, Bernard Ollivier, Michel Magot

**Affiliations:** ^1^Equipe Environnement et Microbiologie, Institut des Sciences Analytiques et de Physico-Chimie pour l'Environnement et les Matériaux (IPREM UMR 5254), Université de Pau et des Pays de l'AdourPau, France; ^2^Mediterranean Institute of Oceanology (MIO), Aix-Marseille Université, Université du Sud Toulon-Var, CNRS/INSU, IRD, UM 110Marseille, France

**Keywords:** *Desulfotomaculum*, deep subsurface, geomicrobiology, sulfate-reduction, lithoautotrophy, sporulation

## Abstract

Gram-positive spore-forming sulfate reducers and particularly members of the genus *Desulfotomaculum* are commonly found in the subsurface biosphere by culture based and molecular approaches. Due to their metabolic versatility and their ability to persist as endospores. *Desulfotomaculum* spp. are well-adapted for colonizing environments through a slow sedimentation process. Because of their ability to grow autotrophically (H_2_/CO_2_) and produce sulfide or acetate, these microorganisms may play key roles in deep lithoautotrophic microbial communities. Available data about *Desulfotomaculum* spp. and related species from studies carried out from deep freshwater lakes, marine sediments, oligotrophic and organic rich deep geological settings are discussed in this review.

## Introduction

In 1955, Morita and Zobell suggested that life probably does not exist below 7.47 m of the sea-floor (Morita and Zobell, 1955). But now, 60 years later, resurgence of deep biosphere studies have greatly expanded our understanding of life at these depths (Ghiorse and Wilson, 1988; Fliermans and Balkwill, 1989; Gold, 1992; Pedersen, 2000; Guan et al., 2013). Microbes have been shown to survive at depths of 2800 m (Chivian et al., 2008), 3200 m (DeFlaun et al., 2007), or even 5278 m (Szewzyk et al., 1994). Therefore, the deep biosphere becomes the potentially major habitat for prokaryotes although cells concentration remains low (Whitman et al., 1998) and can be highly variable likely because of geological features and sediment composition (Sinclair and Ghiorse, 1989). Although extrapolation is difficult because of the lack of measurements, the only prokaryotic biomass in the terrestrial subsurface has been estimated similar or higher than biomass from terrestrial surface (Gold, 1992; Whitman et al., 1998; Pedersen, 2000). Despite recent interest, current knowledge of the microbiology of the Earth's deep subsurface is still incomplete for many reasons. Studying the deep subsurface is challenging simply because of the extreme physical and economical barriers involved with sampling these formations. This is especially the case when collecting representative samples of deep geological formations to study their native microbial composition. Specific sampling protocols have been developed to limit contamination during sampling, or at least estimate the contamination. These protocols include well cleaning procedures (Basso et al., 2005), flame sterilization of the core exterior (Lever et al., 2006) and contamination tests including chemical tracers, microspheres, cultivation and DNA assays (Colwell et al., 1992; Russell et al., 1992; Smith et al., 2000; Lever et al., 2006). Further measures to prevent contamination of subsurface samples are still needed, as current protocol still allows for some contamination (Santelli et al., 2010; Lever, 2013). It is important to overcome these limitations because the identification and understanding of the deep life is useful for industrial applications (corrosion, well-souring, microbial enhanced oil recovery), environmental clean-up (migration of long-lived radio-nucleides in disposal of radioactive waste, water contamination) as well as gaining knowledge of extreme life. Some scientists argue that the origins of life could be intra-terrestrial and could be especially related to a chemical reaction altering low-silica ultramafic rocks, so-called serpentinization, producing high amount of hydrogen necessary as energy for deep microbial life (Ménez et al., 2012; Schrenk et al., 2013). From studies on this kind of environment (Schrenk et al., 2013), but also in many other subsurface systems (Ishii et al., 2000; Magot et al., 2000; Klein et al., 2001; Ollivier and Magot, 2005; Brazelton et al., 2006; Basso et al., 2009), it seems to emerge that Gram-positive, spore-forming sulfate reducers are common inhabitants of the deep subsurface, and may play an essential role in the dynamics of deeply buried anaerobic bacterial communities. This review aims to summarize available data and discuss how the main physiological traits of members of this bacterial group allow their adaptation to such environments.

### The *desulfotomaculum* genus

Most of the data discussed in the present review concern *Desulfotomaculum* species. Nevertheless, sulfate-reducing Gram-positive *Bacteria* also include *Desulfosporosinus* spp., *Desulfovirgula* sp., *Desulfispora* sp. and the candidate species “*Desulforudis audaxviator.”* The few available data dealing with the presence of these three other genera in the deep subsurface will be discussed later in this paper when necessary.

*Desulfotomaculum* spp. are anaerobic bacteria using sulfate as terminal electron acceptor, which is reduced to sulfide. They are members of the phylum *Firmicutes*, class *Clostridia*, order *Clostridiales* and family *Peptococcaceae* (Kuever and Rainey, 2009). When grown in pure cultures, *Desulfotomaculum* cells are straight or curved rods of dimensions 0.3–2.5 × 2.5–15 microns with rounded or pointed ends. Moreover, *Desulfotomaculum* spp. are spore-forming bacteria with central to terminal round or oval spores, often causing swelling of the cells. The genus is composed of 30 validly described species and one subspecies to date. Among these, 17 are thermophilic or moderately thermophilic, 3 are halophilic and one is alkaliphilic.

Beside sulfate, some species described to date can use other sulfur-containing inorganic compounds including thiosulfate, sulfite, and elemental sulfur as terminal electron acceptors (Kuever and Rainey, 2009). While the disproportionation of sulfur compounds (e.g., thiosulfate, elemental sulfur) has been demonstrated to frequently occur among the members of the *Deltaproteobacteria* class (Lovley and Phillips, 1994; Finster, 2008), there are only two reports on the ability of *Desulfotomaculum* spp to perform thiosulfate disproportionation. They include *D. thermobenzoicum* (Jackson and Mcinerney, 2000) and *D. nigificans* (Nazina et al., 2005). In addition, *Desulfotomaculum reducens* was shown to use metals [e.g., Mn(IV), Fe(III), U(VI) or Cr(VI)] as terminal electron acceptors (Tebo and Obraztsova, 1998). Numerous species including *D. geothermicum*, *D. salinum* and *D. kuznetsovii* (Daumas et al., 1988; Nazina et al., 1989, 2005), oxidize H_2_, but also organic acids and long chain fatty acids. Some *Desulfotomaculum* spp. may grow autotrophically on hydrogen (Daumas et al., 1988; Nazina et al., 1989; Tasaki et al., 1991) or may oxidize it by reducing CO_2_ into acetate. This metabolic process known as homoacetogenesis was only demonstrated for *D. gibsoniae* (Kuever et al., 1999), however, it has only been investigated in a few species. Several species use carbohydrates as electron donors. They include *D. putei*, *D. carboxydivorans*, and *D. geothermicum* (Daumas et al., 1988; Liu et al., 1997; Parshina et al., 2005). Substrates are either completely oxidized to CO_2_ or incompletely oxidized to acetate. In the absence of sulfate, some species can also grow by fermentation of glucose, fructose or pyruvate. It was also recently shown that *Desulfotomaculum* sp. Ox39 utilized aromatic hydrocarbons (e.g., toluene, m-xylene, o-xylene) as carbon and energy sources (Morasch et al., 2004). This ability to oxidize monoaromatic hydrocarbons has also been reported for other undescribed members of this genus (Berlendis et al., 2010).

### The deep biosphere

Different definitions of the extent of the deep subsurface (Fliermans and Balkwill, 1989; Sinclair and Ghiorse, 1989; Pedersen, 1993) have proposed an upper limit of between 10 and 100 m below the ground or seabed. From our point of view, deep environments should rather be defined as subsurface settings isolated from the current and direct influence of surface environments. Water column or surface sediments thus do not fit our definition, however, they are also discussed below in certain instances where they can be considered as gateway environments linked to truly deep environments, i.e., the deep geological formations, through sedimentation.

The deep environments show great differences regarding nutrient availability. Some of them are rich in organic matter which can be used as electron donors and/or carbon sources by the microorganisms while others are oligotrophic environments where carbon dioxide and hydrogen are sometimes the only carbon and energy sources available. These different environmental conditions are discussed separately below.

One important characteristic of subsurface environments is the availability of hydrogen to be used by autotrophic microorganisms. It can originate from the low but significant radioactivity of rocks by radiolysis of water, or from anaerobic mineral reactions (Stevens and Mckinley, 1995; Schrenk et al., 2013). There is also a low but constant flux of hydrogen from the Earth mantle to surface. Its presence is favorable to the development of autotrophic and homoacetogenic anaerobic microorganisms in the subsurface. Nevertheless, this is particularly important in oligotrophic environments. Where organic matter is abundant, like in sediments, even chemolithotrophs rely on energy that is released by the chemoorganotrophic breakdown of photosynthetically produced organic matter—nonetheless they are present and active.

## Distribution of *desulfotomaculum* spp. in different subsurface environments

*Desulfotomaculum* spp. have been isolated and detected by molecular approaches in various ecosystems including the bovine rumen, feces, rice fields, but also in subsurface environments including freshwater and marine sediments, mines, oil reservoirs, and aquifers (Magot et al., 2000; Kaksonen et al., 2006; Ollivier et al., 2007; Wang et al., 2008). The frequent discovery of spore-forming sulfate reducers in deep environmental samples suggests their particular adaptation to extreme conditions in the subsurface, which could involve (i) sporulation, (ii) the ability to grow autotrophically (Klemps et al., 1985; Kotelnikova and Pedersen, 1997), and (iii) the ability to grow at elevated temperatures.

### Deep fresh water lakes

The deep waters of lakes are not, strictly speaking, deep subsurface environments as defined in the introduction. However, some published work shows how bacteria can use this gateway to the subsurface thanks to their physiological adaptation to changing environments, and therefore these environments deserve to be mentioned in this review.

The surface and deep waters of the meromictic Lake Gek-Gel in Azerbaijan are mixed less than once a year, resulting in the formation of a deep anoxic zone (Karnachuk et al., 2006). *Desulfotomaculum* spp. were detected in the water column of this lake at depth below 31 m where there is no more dissolved oxygen. In addition, the proportion of *Desulfotomaculum* spp. in the water column determined by FISH increased with depth (0% of total microorganisms at 10 m 7% at 16 m, and 31% at 70 m), while species of the genera *Desulfovibrio* and *Desulfomicrobium* (*Deltaproteobacteria*) appear to follow a reverse trend. These results thus suggested that *Desulfotomaculum* spp. play a major role in the anoxic zone of the lake. Moreover, it is unlikely that the chemical treatment for FISH applied to the samples was efficient enough to allow the detection of *Desulfotomaculum* spores. It can therefore be assumed that the overall number of *Desulfotomaculum* could potentially be even higher than reported.

Strains related to the genus *Desulfotomaculum* have also been isolated from sediments of the oligotrophic Lake Stechlin located in the north of Berlin, Germany (Sass et al., 1998). This is a dimictic lake where the deep anoxic zone and the oxic surface layer mix twice a year, once in winter and the other in summer. In this study, 27 bacterial strains were isolated from sediments collected near the shore and approximately 30 m deep in the lake. Interestingly, all isolated Gram-positive bacteria were related to the spore-forming genera *Desulfotomaculum* and *Sporomusa* (Möller et al., 1984). They represented nearly 40% of all bacteria isolated at depth in this lake. Another study on these sediment samples again suggested that *Desulfotomaculum* isolates represented the dominant microbial community within the deepest layers of sediments (Sass et al., 1997). These results are consistent with data reported by Stahl et al. (2002) suggesting that Gram-positive sulfate-reducing bacteria (SRB) are the main sulfidogenic microorganisms in deep environments.

These two studies illustrated that not only sediments, but also lakes waters can be habitats for anaerobic microorganisms if they are deep enough to have a permanent or semi-permanent anoxic deep zone. Sass et al. (1997) stated that the presence of *Desulfotomaculum* spp. in fresh waters is not surprising given the low nutrient intake in these niches. Indeed, as mentioned above, *Desulfotomaculum* cells can sporulate and thus survive long deprivation periods. However, the spore-forming ability may not be the only reason for the resistance of these microorganisms in these environments since only 0.5% of *Desulfotomaculum* cells sampled in Lake Stechlin were shown to be present as spores and survived pasteurization (Sass et al., 1997). In contrast, Bak and Pfennig (1991) showed earlier that 50% of the *Desulfotomaculum* found in Lake Constance (on the border between Switzerland and Germany) were in the sporulated form. Nevertheless, it seems that such differences are difficult to explain until being able to link sporulation with the detailed physico-chemical conditions which could have influenced it in the studied lakes.

In this respect, the distribution of *Desulfotomaculum* in deep lakes can be explained by the adequacy of their physiological characteristics to particular and changing physicochemical conditions. *Desulfotomaculum* spp. are generally predominant in anoxic environments where sulfate concentrations are low (Ingvorsen et al., 1981; Widdel, 1988; Leloup et al., 2006). The selection of *Desulfotomaculum* species entering the sedimentation process should possibly also results from their metabolic capabilities. Castro et al. (2002) reported that complete oxidizers represented 95% of the *Desulfotomaculum* spp. in several human-impacted environments, whereas in pristine environments, with significantly lower concentrations in total phosphorus and carbon, all *Desulfotomaculum*-related sequences were related to incomplete oxidizing species. From these results authors hypothesized that the versatility of complete oxidizers allows them to proliferate in substrate-rich environments and those incomplete oxidizers were more adapted to use specific nutrients in pristine environments. These conditions correspond to those found at the bottom of deep freshwater lakes, but specific studies dealing with substrate oxidation in these environments are lacking. Anyway, we can speculate that during water mixing, although nutrients are renewed in depth, growth of *Desulfotomaculum* members should be limited due to the presence of oxygen. Once the favorable redox conditions are back in deep water and shallow sediments, Gram-positive bacterial spores can germinate and growth of selected *Desulfotomaculum* strains starts again.

### Deep subsurface environments

Microbiological studies of deep subsurface samples are scarce. Sampling deep geological layers is most often closely linked to industrial activities, although specific national or international scientific programs dedicated to the study of the deep subsurface do exist. The reason is obviously that the cost of drilling at great depths can generally be assumed only if there is an industrial interest. Most of these studies are thus related to the industrial exploitation of the subsurface, including mining, oil production, aquifers, or different types of storages in the subsurface (e.g., natural gas, nuclear wastes). Although these environments have many similar physical properties such as temperature and pressure, they can be very different regarding the availability of nutrients.

#### Oligotrophic deep geological settings

Ore mining gives access to very deep geological layers. Microbiological studies carried out in the mines often highlighted the presence of Gram-positive sulfate-reducing *Bacteria* adapted to the *in situ* extreme conditions. In Japan, *D. thermosubterraneum* was isolated from a geothermally active underground mine at a depth of 250 m (Kaksonen et al., 2006). This species is thermophilic (optimum temperature between 60 and 65°C), using a wide range of electron donors and is notably able to grow autotrophically on H_2_. *Desulfovirgula thermocuniculi*, another Gram-positive sulfate-reducer from the domain *Bacteria* was isolated from the same mine. This thermophilic species use H_2_ and various organic acids as electron donors and may ferment pyruvate and lactate (Kaksonen et al., 2007).

Different Gram-positive SRB were detected in the Kamaishi mine (Ishii et al., 2000). Their 16S rRNA gene sequences related to both mesophilic and thermophilic *Bacteria* were retrieved from 400 to 800 m deep anoxic cold water samples (10–15°C). A new examination of these sequence data shows that the closest relatives are *Desulfotomaculum geothermicum* (91% similarity) but also other Gram-positive genera and species including *Desulfosporosinus lacus* (91% similarity), *Desulfosporosinus acidiphilus* (93% similarity), *Desulfosporosinus youngiae* (95% similarity), and *Candidatus Desulforudis audaxviator* (93% similarity). These lineages represent 9 among the 15 phylotypes characterized by DGGE analysis, demonstrating the importance of spore-forming bacteria in this environment. However, lack of data on vegetative vs. sporulated cells limits the interpretation regarding the activity of these bacteria under the mesothermic *in situ* conditions. Although the authors mentioned that “it remains to be solved whether *Desulfotomaculum* spp. existed in spores or vegetative cells in the groundwater,” only vegetative cells were probably taken into consideration considering the DNA extraction protocol used. Necessary improvements of spore DNA extraction protocols are discussed later in this review.

The importance of Gram-positive SRB in deep environments is also emphasized by data from the Toyoha mine (Nakagawa et al., 2002). The dsrAB gene sequences from high temperatures samples (50–70°C) indicated the presence of microorganisms closely related to two autotrophic species, *D. kuznetsovii* and *D. thermocisternum*. The *Desulfotomaculum* species harboring these genes could thus possibly live autotrophically in the nutrient-limited environment of the mine, thus participating to a subsurface lithoautotrophic microbial ecosystem (SLiME) as described by Stevens and Mckinley (1995).

Prokaryotes were also found in much deeper environments, as in a South Africa mine where two genera, *Desulfotomaculum* and *Methanobacterium*, represented the dominant microflora (Moser et al., 2005). Clone libraries of 16S rRNA, sulfite reductase (dsrAB) and methyl-coenzyme M reductase genes were constructed from 3200 to 3300 m deep samples. At 3200 m *Desulfotomaculum* spp. represented 100% of the bacterial clones (16S rRNA and dsrAB) and *Methanobacterium* 100% of archaeal clones. At 3300 m the profiles are very similar, with of 98% and 97% respectively. Unfortunately the results obtained by this type of approach did not allow the estimation of the *Bacteria*/*Archaea* balance, since two independent clone libraries were constructed. However, it was assumed that both groups coexist without any of them becoming permanently dominant, due to the specific conditions prevailing in this environment. Unlike the conditions observed in the seabed (Parkes et al., 2000; D'hondt et al., 2004) or in the shallow continental sediments, hydrogen is not a limiting electron donor in this environment. Thus, Moser et al. (2005) argue that *Desulfotomaculum* populations are limited by the low sulfate content, whereas the *Methanobacteriaceae* populations are limited by the low bioavailability of carbonates used as electron acceptors due to high pH and high concentration of Ca^2+^ and the low energy supplied by methanogenesis in these conditions. The presence of these two microbial genera has also been reported in the Lost City samples (Gerasimchuk et al., 2010). Beside microbial diversity, temperature (60–75°C), pH (9–11) and high concentrations of CH_4_ and H_2_ were similar characteristics of both environments.

The most extreme case of microbial adaptation to depth was described in the 2.8 km underground Mponeng gold mine in South Africa. Chivian et al. (2008) revealed an ecosystem containing a single (or extremely dominant) microbial population, *Candidatus Desulforudis audaxviator*. This uncultivated Gram-positive putative sulfate reducer, whose closest relative is *Pelotomaculum thermopropionicum*, is only known by the sequence of its genome, retrieved from a metagenomic study of a fault water sample. Genes necessary for autotrophic life and sulfate reduction were shown to be present, in agreement with the adaptation of *D. audaxviator* to its deep environment. Its genetic package also includes sporulation genes. In contrast to studied *Desulfotomaculum* spp., the genome of *D. audaxviator* does not encode a complete system of resistance to oxygen, suggesting a long-term isolation of the species.

Few studies of the microbial diversity of deep aquifers have been published, but deep water-bearing formations are potential ecological niches for *Desulfotomaculum* and *Desulfosporosinus* species (Colwell et al., 1997; Detmers et al., 2001, 2004; Stahl et al., 2002). Studies of a pristine aquifer have shown a presence of *Desulfotomaculum* spp. in a 120 m deep geological formation (Detmers et al., 2004). Eight strains were isolated from these samples including 6 *Firmicutes*, among which 4 strains belong to the genus *Desulfotomaculum*. The authors suggested that these microorganisms were indigenous to this type of geological formation. Other deep aquifer samples were studied by Itavaara et al. in Finland (2011). Clone libraries from three different depth intervals (0–100 m, 900–1000 m and 1400–1500 m) showed that the proportion of *Desulfotomaculum* spp. and *Desulfosporosinus* spp. increased with depth. They represented up to 23% of the sequences obtained at 1500 m and only 8.5% at 1000 m. It is interesting to note that spore-forming Gram-positive *Clostridia* (the bacterial class including *Desulfotomaculum* spp. and *Desulfosporosinus* spp.) in the clone library account for 75% of the sequences obtained at depth of 1500 m.

More recently, *Desulfotomaculum* spp. were shown to represent 25% of the 16S rRNA sequences retrieved from a 800 m-deep aquifer of the Paris Basin (Basso et al., 2009). Four phylotypes related to *D. putei* (97% similarity), *D. geothermicum* (95% similarity), *D. arcticum* (95% similarity) and *D. kuznetsovii* (91% similarity) were characterized. Although many *Desulfotomaculum*-related sequences were present in the clone library, cultivation experiments were not successful, thus highlighting the difficulty to grow and isolate spore-forming sulfate reducers among fast growing other bacterial types (e.g., *Desulfovibrio* spp.).

#### Deep environments rich in organic matter

Long after the pioneering work of Bastin (Bastin et al., 1926) which preceded the discovery of the genus *Desulfotomaculum*, and particularly since the early 1980s, microbiological studies of deep oilfields production waters have led to a better understanding of the adverse effects of sulfate-reducing microorganisms in the oil industry: corrosion of metal equipment, lowering of the permeability of geological formations by precipitation of iron sulfides, hydrogen sulfide harmful to the health of employees, “souring” of reservoirs, and decrease of the crude oil value (Magot, 2005; Hubert, 2010; Gieg et al., 2011; Kakooei et al., 2012). These studies highlighted the presence of many SRB including those pertaining to the genus *Desulfotomaculum* in geographically distant very deep geological formations (Magot et al., 2000; Ollivier and Magot, 2005; Ollivier and Alazard, 2010). Particular attention was paid to the North Sea oilfields (Rosnes et al., 1991; Nilsen et al., 1996a,b; Leu et al., 1998; Gittel et al., 2009) where the presence of *Desulfotomaculum* species in reservoirs between 2 and 4 km below the seabed was consistently reported. The presence of *Desulfotomaculum* spp. was also reported in oilfield production waters in France (Tardy-Jacquenod et al., 1996), China (Liu et al., 2008; Lan et al., 2011), India (Agrawal et al., 2010), and in numerous other locations on the planet (Ollivier and Magot, 2005).

Recently, Guan et al. (2013) showed that in four distant Chinese oil reservoirs SRB diversity was low with a predominance of *Desulfotomaculum* species and lower representation of *Deltaproteobacteria*. A positive correlation was observed between the presence of *Desulfotomaculum* phylotypes and depth. At 480 m (28°C), 802 m (37°C) and 1300 m (62°C), *Desulfotomaculum* 16S rRNA gene sequences represented 10%, 40% and 70% of the total SRB sequences, respectively. At 1490 m (58°C) *Desulfotomaculum* 16S rRNA gene sequences represented 80% of total SRB phylotypes. Other correlations obtained by statistical tests with e.g., sulfate, acetate and propionate concentrations were not as strong and deserve further investigations.

Four validly described *Desulfotomaculum* species were originally isolated from oil field waters: *D. kuznetsovii*, *D. salinum*, *D. thermocisternum* and *D. halophilum* (Nazina et al., 1989, 2005; Nilsen et al., 1996b; Tardy-Jacquenod et al., 1998, respectively). Interestingly, both complete and incomplete substrates oxidizers were described (Table 1). Assuming that these species are truly indigenous bacteria of the geological formation and not contaminants or simply spores, this observation could be analyzed as contradictory with the hypothesis of Castro et al. (2002) suggesting a better adaptation of complete oxidizing *Desulfotomaculum* to substrate-rich environments. But the eutrophic environments of the Everglades studied by this team are extremely different from oil reservoirs where crude oil and its hydrocarbon components are not easily utilizable substrates under anaerobic conditions.

**Table 1:**
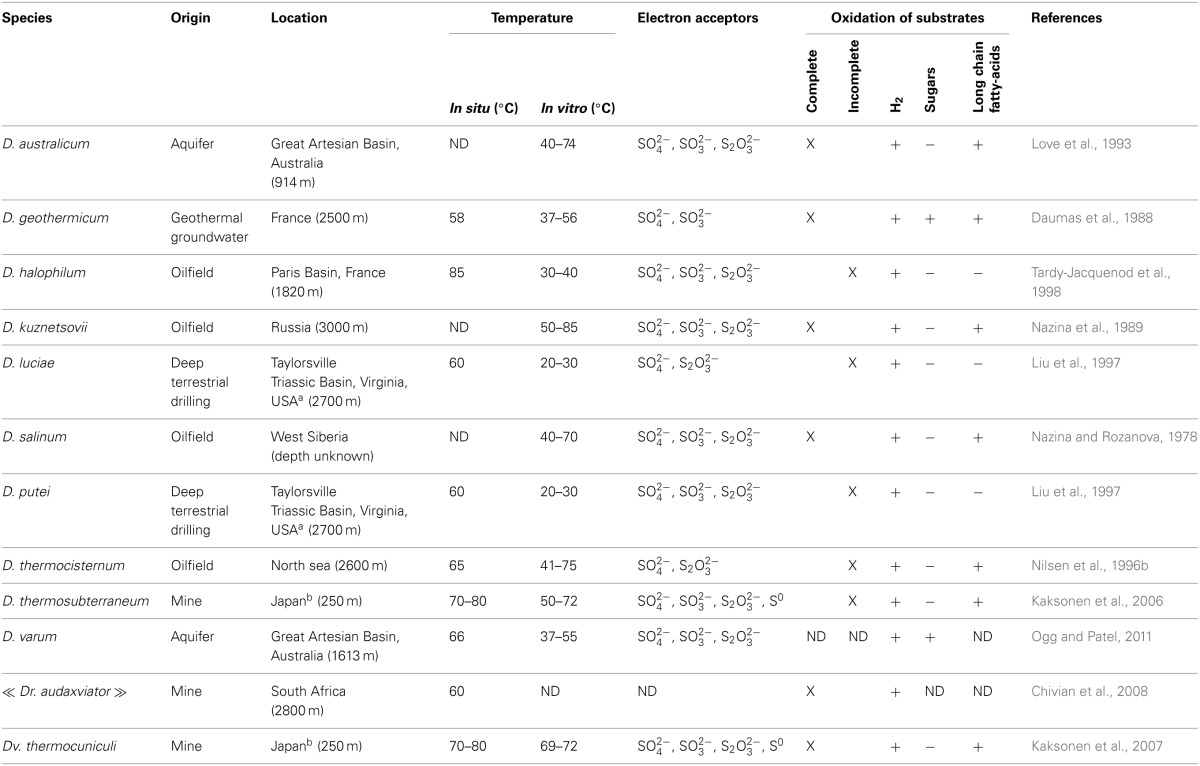
**Main characteristics of *Desulfotomaculum* species isolated from the deep subsurface**.

*D, Desulfotomaculum; Dr, Desulfoviridis; Dv, Desulfovirgula; Ds, Desulfosporosinus; ND, Not Determinated*.

*^a^ and ^b^ indicate species collected at the same site*.

### Marine seabed

In the Atlantic Ocean, microorganisms related to the genus *Desulfotomaculum* were generally not found at deep-sea hydrothermal vents (Ollivier et al., 2007). Nevertheless, the Lost City hydrothermal field near the mid-Atlantic ridge at a depth of 900 m represents a specific unusual case, where Brazelton et al. (2006) demonstrated that the cloned 16S rRNA genes related to *D. halophilum* (88% identity) and *D. alkaliphilum* (92% identity) represented the only sulfate-reducing *Bacteria* identified in this environment. These results are in agreement with those of Gerasimchuk et al. (2010), as the cloned 16S rRNA genes distantly related to *D. alkaliphilum*, *D. halophilum* and *D. thermocisternum* were found in sediments collected from the same site (with sequence identities between 88 and 91%). Furthermore, the potential ability to reduce sulfate was revealed by the detection of genes encoding the dissimilatory sulfate reductase (*dsr*AB) enzyme. These genes sequences were related with those of incomplete oxidizers *Desulfotomaculum* spp. as described by Klein et al. (2001). These informations are consistent with the limitation of carbon observed on the Lost City chimneys, where oxidized organic compounds originate from synthrophic anaerobic oxidation of methane (Bradley et al., 2009).

Mineralization of sedimentary organic matter occurs at the seabed where SRB play a major role in the carbon and sulfur cycles (Jørgensen, 1977). Many studies have shown the presence of Gram-positive SRB in deep marine environments and in shallow sediments under deep seawater columns (Reed et al., 2002; Zhuang et al., 2003; Teske, 2006). Such microorganisms seem to predominate among all sulfate reducers, as shown in the sediments of the 1900 m deep West Pacific Warm pool, where they represented 54% of the sulfate-reducing population at 1–12 cm depth (Wang et al., 2008). However, FISH counts showed that SRB only constitute 0.3–2% of active microorganisms in this ecosystem.

The presence of spores in surface sediments (0–25 cm deep) from the Arctic Ocean was revealed by endospore germination experiments (Hubert et al., 2009). Thermophilic *Firmicutes* including *Desulfotomaculum* spp. were identified in 16S rRNA genes clone libraries. More recently, Hubert et al. (2010) showed that spores of thermophilic microorganisms scattered on the Arctic seabed mineralize complex organic matter at 50°C *in vitro*. This activity was catalyzed via extracellular enzymatic hydrolysis, fermentation and sulfate reduction. Four distinct *Desulfotomaculum* phylotypes were identified. The most prominent was related to *D. halophilum* (91% identity), whereas three other lineages were related to *D. geothermicum*, *D. reducens*, and *D. thermosapovorans* (95–96% identity).

Very few information on the microbial populations in deep marine sediments are available, obviously because of the difficulty to collect samples through drilling under a deep water column. A metagenomic analysis of sediments of the Peru Margin (Ocean Drilling Program Leg 201, site 1229D) at depth from 1 to 50 m below the seafloor (mbsf) showed a minor contribution of *Firmicutes* to the microbial diversity, but did not give specific informations about *Desulfotomaculum* or related genera (Biddle et al., 2008). A metatranscriptomic study of samples from the same site at depths up to 159 mbsf was recently published. It showed that *Firmicutes*, and to a lesser extent *Deltaproteobacteria*, are the dominant phyla expressing *dsr* transcripts (sulfite-reductase, involved in sulfate respiration) at 5 and 30 mbsf. At greater depth, below the sulfate-methane transition zone where sulfate concentration is under detection limit, expression of *dsr* genes is linked to the activity of *Gammaproteobacteria* (Orsi et al., 2013). These data suggest that Gram-positive SRB are mainly active in shallow sediments. Interestingly the recently published culture-based study of sediments of the Juan De Fuca Ridge (IODP Site U1301) gave similar indications (Fichtel et al., 2012). Isolates of *Desulfosporosinus lacus* and *Desulfotomaculum* spp. were only retrieved from the upper 9.1 mbsf sediments, whether *Desulfovibrio* and *Desulfotignum* isolates were cultivated from deeper sediments up to 262 mbsf. Nevertheless, a culture-based study of ODP sites, 3 in the Open Pacific and 4 on the Peru Margin, including the 1229 site, did not reveal the presence of sulfate-reducing *Firmicutes* in samples collected at depth of 1 to 420 mbsf (D'hondt et al., 2004).

The few available studies summarized above suggest that the spore-forming SRB are found and are active only in the most superficial marine sediments, but are absent in depth, especially below the sulfate-methane transition zone. However, since too few studies of this type are currently available, it is premature to generalize these results.

## Physiological advantages of spore-forming sulfate reducers for their adaptation to subsurface environments

### Sporulation

One can speculate that the presence of most microorganisms inhabiting the deep subsurface is the outcome of a gradual burial of surface organisms over geological times. During this long term process, the physico-chemical conditions gradually change according to geological constraints (rock type, depth, pressure, thermogenic processes …) and organic matter is degraded or transformed through biological or abiotic processes. Spore formation is putatively one of the key characteristics of sulfate-reducing or fermentative *Clostridiales* helping them to survive transient periods of nutrient deprivation, unfavorable temperature or redox conditions during burial, and consequently allowing deep subsurface colonization (Kuever and Rainey, 2009). These “survival periods” could be extremely long, since it is well-documented that endospore-forming bacteria, such as *Desulfotomaculum*, remain viable in a wide variety of environments unfavorable for growth possibly during thousands or millions of years, like in amber (Cano and Borucki, 1995), halite crystals (Vreeland et al., 2000) or marine manganese nodules (Nealson and Ford, 1980). However, even if large amounts of spores (10^7^ endospores per cm^3^) can be found in several million year-old samples (Lomstein et al., 2012), many authors wonder about the possibility of spores activation and germination without DNA maintenance activity for very long periods (Lindahl, 1993; Johnson et al., 2007). A recent study of sediments from the Baltic Sea by De Rezende et al. (2013) used an MPN technique with a specific SRB culture medium. Interestingly, it was shown that the number of spores of thermophilic *Desulfotomaculum* decreased from 10^4^ spores per cm^3^ of sediment at the surface to one spore per cm^3^ at 6.5 m depth. Given that the sediment age at the deeper level is approximately 4500 years, the half-life of spores has consequently been estimated to be about 400 years. Nevertheless, if the survival of spores is as short as de Rezende and colleagues estimated, it seems difficult to explain the abundant presence of *Desulfotomaculum* in geological settings located several kilometers deep. Taking into account these contrasting results, further investigations are needed to better understand the distribution of *Desulfotomaculum* spp. between shallow and deep sediments, and connections between these different populations. On the other hand, microorganisms could also survive with low metabolic activity with generation times in the range of 1 × 10^3^ to 3 × 10^3^ years (Whitman et al., 1998; Lomstein et al., 2012). Sporulation could be periodically necessary to overcome extreme energy limitation because of a complete depletion of local nutrient supply (Fredrickson and Balkwill, 2006), or to facilitate passive dispersal (Isaksen et al., 1994; Jørgensen and Boetius, 2007; Hubert et al., 2009; De Rezende et al., 2013). At the end of the sedimentary process, extreme situations can be reached as in the case of the putative spore-forming *Candidatus Desulforudis audaxviator* that became the single bacterial autonomous species in its deep environment (Chivian et al., 2008). The detection of *Desulfotomaculum* spp. in northern sea sediments also illustrate how spore formation could be an advantage for the dissemination of microorganisms originating from the deep subsurface in surface environments, which may finally return to shallow sediments. *Desulfotomaculum arcticum* was isolated from sediments of Nordfjorden (under a 100 m deep water column) on the west coast of Svalbard in the Arctic Ocean (Vandieken et al., 2006). Since this species grows optimally at 42°C, it was suggested that it was present as spores in the cold sediments. *D. arcticum* share metabolic traits with most of species of the genus, including the use of H_2_ as potential energy source. Two hypotheses were proposed to explain the presence of thermophilic spores in the cold seabed of the Arctic Ocean. The first refers to the oceanic crust fluids that harbor microbial communities including *Firmicutes* species (Cowen et al., 2003). These microorganisms might be transported to the oceanic ridge before being dispersed into the ocean via hydrothermal vents such as those of the Lost City (Brazelton et al., 2006). The second hypothesis suggests that spores come from oilfields in which *Desulfotomaculum* spp. have often been detected (see below) either from produced oil reservoirs (Magot et al., 2000), or from natural seeps. The physiologic traits of thermophilic bacteria found in the Arctic Sea support the idea of their adaptation to the hot subsurface conditions. It is thus suggested that the ocean floor would be connected to the oceanic crust and the oilfields by oceanic fluids. A similar idea has already been suggested by Stetter et al. (1993), assuming that hyperthermophiles from hydrothermal vents should have been transported into oil reservoirs by seawater injection.

### Physiology and metabolism

It is noteworthy that many *Desulfotomaculum* spp. isolated from subsurface environments are autotrophic microorganisms. As indicated above, this metabolic ability allows them to take advantage of the recognized ubiquitous resource in hydrogen in the deep subsurface (Pedersen, 1997; Lin et al., 2005). Moreover, some of these SRB may also perform a homoacetogenic metabolism by oxidizing hydrogen and reducing CO_2_ to acetate. There are more and more convincing results that such metabolism plays an important role in carbon cycling in the deep biosphere (Lever, 2011; Oren, 2012). In this respect, the delivery of acetate resulting from this metabolic process in the deep biosphere in particular, can be beneficial to the overall microbial community with a peculiar emphasis for (i) acetoclastic methanogenic archaea which have been recovered from the deep biosphere and (ii) other hydrogenotrophic SRB, or hydrogen-oxidizing methanoarchaea inhabiting subsurface ecosystems and requiring an organic carbon source for their growth. Only by taking into account these peculiar metabolic features of deep-living *Desulfotomaculum* spp. (autotrophic way of life and homoacetogenesis), it seems evident that they could be a significant, even major component for SLiME. Evidence has been provided at many occasions in the deep biosphere that sulfate-reducers coexist with homoacetogenic bacteria and methanogenic archaea. This is particularly true in oligotrophic ecosystems (e.g., deep granitic aquifers) (Pedersen, 1997), but also in organic compounds rich ecosystems (e.g., oil reservoirs) (Magot et al., 2000; Ollivier and Alazard, 2010) where these three prokaryotic groups have been recovered many times by cultural and molecular approaches. In the former ecosystems, hydrogen recovered from biological (fermentative process) or abiotic processes (e.g., radiolysis, mineral reactions, volcanic activity) has been recognized to drive an intra-terrestrial biosphere (Pedersen, 1997) and hydrogenotrophic autotrophic (i) homoacetogenic bacteria together with (ii) methanogenic archaea have been hypothesized to play a central role to deliver organic material in the deep biosphere (e.g., acetate for homoacetogens, methane for methanogens, and biomass). This makes the *in situ* bioenergetical systems independent of any photosynthetic activity (Pedersen, 1997).

Beside sulfate reduction, we must also consider that *Desulfotomaculum* spp may also use various other sulfur compounds (e.g., thiosulfate, elemental sulfur, sulfite) but also metals and metalloids as terminal electron acceptors (Tebo and Obraztsova, 1998) thus confirming their geomicrobiological significance in the deep biosphere where metals and metalloids in particular are quite widespread. Data on this point are nevertheless scarce, and deserve more investigations. *Desulfotomaculum* species were reported not only to use hydrogen as electron donor, but also a wide range of organic compounds (e.g., sugars, aromatic compounds, long chain fatty acids, organic acids) that may be also available but at less extent in some deep environments rich in organic matters. Clearly, from these metabolic traits that have been established for *Desulfotomaculum* spp, we may expect them to penetrate the deep biosphere, and to adapt to the *in situ* existing extreme physico-chemical conditions in terms of temperature, pressure, or low/rich nutrient availability. However, more exhaustive characterization of the physiology and metabolism of Gram-positive SRB should help to better understand their adaptation to their environments, or even their role in the emergence of different forms of life on Earth. As an example, while many sulfate-reducing members of the *Deltaproteobacteria* are recognized to disproportionate sulfur compounds such as thiosulfate, sulfite or elemental sulfur (Finster, 2008), there are very few evidences of *Desulfotomaculum* to perform such mineral oxidative-reductive processes. This is restricted to thiosulfate disproportionation (e.g., *D. thermobenzoicum* and *D. nigrificans*) and nothing is known so far on the ability of *Desulfotomaculum* spp. to disproportionate elemental sulfur into sulfide and sulfate, such reaction requiring the presence of oxidants (e.g., metal oxides) to be favorable thermodynamically (Finster, 2008). This is quite intriguing and merits further attention by the scientific community since the existence of an oxidative microbial sulfur cycling in which sulfur disproportionation playing a significant role has been postulated (Canfield, 1989; Thamdrup et al., 1993; Jørgensen and Nelson, 2004; Slobodkin et al., 2012) and even recently demonstrated (Riedinger et al., 2010) in the deep biosphere.

## Conclusion and future trends

Interestingly, data collected during the past two decades on the microbiology of deep subsurface environments demonstrate that SRB, particularly for *Desulfotomaculum* species and closely phylogenetically related sulfate-reducing spore-formers, are common in the deep biosphere. Several of their physiological characteristics (e.g., spore formation, anaerobic metabolism, and possible growth under mesophilic or thermophilic conditions) together with their wide metabolic diversity (e.g., use of hydrogen, sugars, alcohols, long chain fatty acids, aromatic compounds) help them to penetrate deeply buried ecological niches, and to adapt to the existing physico-chemical conditions. This is particularly of significance with regard to autotrophic growth of these SRB by possibly oxidizing hydrogen via sulfate or CO_2_ reduction (homoacetogenesis), resulting in the production of sulfide and acetate, respectively. Beside hydrogen oxidation recognized to be of geomicrobiological significance in the deep biosphere, more attention should also be paid to possible involvement of these Gram-positive bacteria in oxidative-reductive processes of inorganic sulfur compounds as native sulfur has been found at many occasions not only in terrestrial but also in subterrestrial ecosystems (Jones et al., 1956; Tritla et al., 2000; Alonso-Azcárate et al., 2001; Ziegenbalg et al., 2010). Such metabolisms shared by many Gram-negative SRB, and rarely demonstrated amongst Gram-positive SRB should be tested more intensively as they may bring also answers to the delivery of organic matter by the latter microorganisms in the deep biosphere from an energy-yielding mineral fermentative process (e.g., sulfur disproportionation). More attention should also be paid to this metabolism more generally since procaryotes involved in it have most probably been of major importance in early bacterial life (Philippot et al., 2007; Finster, 2008).

Beside these metabolic features determining to what extent these bacteria are essential components for life in the subsurface needs, however, further investigations. This should start with the study of a larger number of subsurface samples collected under appropriate conditions (Basso et al., 2005; Kieft et al., 2007; Lever et al., 2013). Particular attention should be paid to the drilling conditions and core recovery, as done in the Integrated Ocean Drilling Projects (IODP) and International Continental Scientific Drilling Program (ICDP), which should logically be among the main providers of biological samples for the study of the deep biosphere. Knowledge of the deep subsurface microbial ecology would benefit from increased research in IODP and ICDP focused on this issue, and from the use of the full potential of specific equipments that can offer these collaborative projects. Samples obtained should be studied by combining culture-based and molecular approaches of microbial diversity. Metagenomic, metatranscriptomic and metabolomic studies may be considered as recent advances in microbial ecology and have fundamentally modified our knowledge of the microbial ecology in many ecosystems and have pointed more precisely the microbial populations of ecological significance. These approaches should help the scientific community in (i) defining more clearly the existing diversity and (ii) understanding the real functioning of Gram-positive SRB, among other bacterial groups, in the deep biosphere. The work of Biddle's group cited above (Orsi et al., 2013) is a good example of the type of new contributions these approaches can bring to our knowledge of the deep biosphere. Readers interested by this topic can refer to recently published reviews for more information (Reith, 2011; Colwell and D'Hont, 2013).

Technical progresses for studying spore-forming microorganisms are also necessary. More specifically, the issue of monitoring spore-forming bacteria, and distinguishing spores from vegetative cells, is important but difficult because the quantification of spores request to apply specific methods. Most current studies of microbial ecology are based on the use of molecular tools whose effectiveness is variable. Crucial steps of DNA extraction and spore coat permeabilization were considered for *Firmicutes* such as *Bacillus anthracis* (Green et al., 1985; Luna et al., 2003; Thomas et al., 2013), but not for *Firmicutes* such as *Desulfotomaculum* in environmental studies. Actually, the spore coats have to be broken without DNA degradation to allow subsequent extraction for PCR amplification. Although like any DNA extraction protocol must be adapted to the sampled environments mainly because of inhibitors such as humic acids, sulfide, and heavy metal, a protocol recently described based on a novel cryogenic-mill-based procedure seems to be promising and could be adapted to various subsurface samples (Alain et al., 2011) and should be tested in particular for the DNA extraction from spores. Primer sets designed for specifically targeting a part of the 16S rRNA or dsrAB genes from *Desulfotomaculum* can be used to study the diversity of this genus (Daly et al., 2000; De Rezende et al., 2013). For FISH experiments, the spore coats have also to be permeabilized to introduce labeled probes. Several studies to quantify *Bacillus* spp. spores in environmental samples have been successfully carried out (Fischer et al., 1995; Felske et al., 1998; Filion et al., 2009). Interesting results have been obtained when spores were heated for 30 min at 80°C with 50% ethanol and allowed the using of the double-color FISH method for *Bacillus anthracis* spores (Weerasekara et al., 2013). There is a need to test and adapt similar protocols to *Desulfotomaculum* spp. and deep subsurface environmental samples. Currently, best results are obtained through sample pasteurization followed by culture-based (isolation, MPN) and molecular (qPCR, FISH) studies as shown before, even if germination and culture media should probably be still optimized for each specific case. Although such approaches would not allow the *in situ* abundance of the organisms to be determined as these molecular methods (qPCR, FISH) would very likely be being applied to vegetative cells in a post-germination growth phase. On the other hand, indirect techniques must not be neglected. Fichtel et al. (2007) used the quantification of dipicolinic acid, a spore core-specific compound, to estimate spore amounts in cultures and sediments samples. More recently, a method based on this work was described by Lomstein and Jørgensen (2012) while another technique based on muramic acid quantification, a unique building block in cell walls of both bacteria and endospores, was developed by Lomstein et al. (2012). An important limitation of this method is that it does not allow assigning spores to any bacterial species or genus.

Despite the clear evidence that has been provided in recent years that spore-forming Gram-positive SRB are of ecological relevance in subsurface ecosystems, there are scientific challenges that should be overcome to emphasize their role in the poor and rich organic matter deep biosphere in future studies.

### Conflict of interest statement

The authors declare that the research was conducted in the absence of any commercial or financial relationships that could be construed as a potential conflict of interest.

## References

[B1] Agrawal A. Vanbroekhoven K. Lal B. (2010). Diversity of culturable sulfidogenic bacteria in two oil-water separation tanks in the north-eastern oil fields of india. Anaerobe 16, 12–18 10.1016/j.anaerobe.2009.04.005 19427389

[B2] Alain K. Callac N. Ciobanu M.-C. Reynaud Y. Duthoit F. Jebbar M. (2011). DNA extractions from deep subseafloor sediments: novel cryogenic-mill-based procedure and comparison to existing protocols. J. Microbiol. Methods 87, 355–362 10.1016/j.mimet.2011.09.015 22005039

[B3] Alonso-Azcárate J. Bottrell S. H. Tritlla J. (2001). Sulfur redox reactions and formation of native sulfur veins during low grade metamorphism of gypsum evaporites, Cameros Basin (NE Spain) Chem. Geology 174, 389–402 10.1016/S0009-2541(00)00286-2

[B4] Bak F. Pfennig N. (1991). Sulfate-reducing bacteria in littoral sediment of lake constance. FEMS Microbiol. Ecol. 8, 43–52 10.1111/j.1574-6941.1991.tb01707.x

[B5] Basso O. Lascourrèges J. F. Jarry M. Magot M. (2005). The effect of cleaning and disinfecting the sampling well on the microbial communities of deep subsurface water samples. Environ. Microbiol. 7, 13–21 10.1111/j.1462-2920.2004.00660.x 15643931

[B6] Basso O. Lascourreges J.-F. Le Borgne F. Le Goff C. Magot M. (2009). Characterization by culture and molecular analysis of the microbial diversity of a deep subsurface gas storage aquifer. Res. Microbiol. 160, 107–116 10.1016/j.resmic.2008.10.010 19056488

[B7] Bastin E. S. Greer F. E. Merritt C. Moulton G. (1926). The presence of sulphate reducing bacteria in oil field waters. Science 63, 21–24 10.1126/science.63.1618.21 17838563

[B8] Berlendis S. Lascourreges J. F. Schraauwers B. Sivadon P. Magot M. (2010). Anaerobic biodegradation of BTEX by original bacterial communities from an underground gas storage aquifer. Environ. Sci. Technol. 44, 3621–3628 10.1021/es100123b 20380433

[B9] Biddle J. F. Fitz-Gibbon S. Schuster S. C. Brenchley J. E. House C. H. (2008). Metagenomic signatures of the Peru margin subseafloor biosphere show a genetically distinct environment. Proc. Natl. Acad. Sci. U.S.A. 105, 10583–10588 10.1073/pnas.0709942105 18650394 PMC2492506

[B10] Bradley A. S. Hayes J. M. Summons R. E. (2009). Extraordinary ^13^C enrichment of diether lipids at the lost city hydrothermal field indicates a carbon-limited ecosystem. Geochim. Cosmochim. Acta 73, 102–118 10.1016/j.gca.2008.10.005

[B11] Brazelton W. J. Schrenk M. O. Kelley D. S. Baross J. A. (2006). Methane-and sulfur-metabolizing microbial communities dominate the Lost City hydrothermal field ecosystem. Appl. Environ. Microb. 72, 6257–6270 10.1128/AEM.00574-06 16957253 PMC1563643

[B12] Canfield D. E. (1989). Sulfate reduction and oxic respiration in marine sediments: implications for organic carbon preservation in euxinic environments. Deep. Sea. Res. A. 36, 121–138 10.1016/0198-0149(89)90022-8 11542177

[B13] Cano R. J. Borucki M. K. (1995). Revival and identification of bacterial spores in 25-to 40-million-year-old Dominican amber. Science 268, 1060–1064 10.1126/science.7538699 7538699

[B14] Castro H. Reddy K. Ogram A. (2002). Composition and function of sulfate-reducing prokaryotes in eutrophic and pristine areas of the Florida Everglades. Appl. Environ. Microb. 68, 6129–6137 10.1128/AEM.68.12.6129-6137.2002 12450837 PMC134442

[B15] Chivian D. Brodie E. L. Alm E. J. Culley D. E. Dehal P. S. Desantis T. Z. (2008). Environmental genomics reveals a single-species ecosystem deep within earth. Science 322, 275–278 10.1126/science.1155495 18845759

[B16] Colwell F. Stormberg G. Phelps T. Birnbaum S. Mckinley J. Rawson S. (1992). Innovative techniques for collection of saturated and unsaturated subsurface basalts and sediments for microbiological characterization. J. Microbiol. Methods 15, 279–292 10.1016/0167-7012(92)90047-8

[B17] Colwell F. S. D'Hont S. (2013). Nature and extent of the deep biosphere. Rev. Mineral Geochem. 75, 547–574 10.2138/rmg.2013.75.17

[B18] Colwell F. S. Onstott T. C. Delwiche M. E. Chandler D. Fredrickson J. K. Yao Q. J. (1997). Microorganisms from deep, high temperature sandstones: constraints on microbial colonization. FEMS Microbiol. Rev. 20, 425–435 10.1111/j.1574-6976.1997.tb00327.x

[B19] Cowen J. P. Giovannoni S. J. Kenig F. Johnson H. P. Butterfield D. Rappé M. S. (2003). Fluids from aging ocean crust that support microbial life. Science 299, 120–123 10.1126/science.1075653 12511653

[B20] D'hondt S. Jørgensen B. B. Miller D. J. Batzke A. Blake R. Cragg B. A. (2004). Distributions of microbial activities in deep subseafloor sediments. Science 306, 2216–2221 10.1126/science.1101155 15618510

[B21] Daly K. Sharp R. J. McCarthy A. J. (2000). Development of oligonucleotide probes and PCR primers for detecting phylogenetic subgroups of sulfate-reducing bacteria. Microbiology 146, 1693–1705 10878133 10.1099/00221287-146-7-1693

[B22] Daumas S. Cordruwisch R. Garcia J. L. (1988). *Desulfotomaculum geothermicum* sp. nov., a thermophilic, fatty acid-degrading, sulfate-reducing bacterium isolated with H2 from geothermal ground-water. A. Van. Leeuw. J. Microb. 54, 165–178 10.1007/BF00419203 3395110

[B23] De Rezende J. R. Kjeldsen K. U. Hubert C. R. Finster K. Loy A. Jørgensen B. B. (2013). Dispersal of thermophilic *Desulfotomaculum* endospores into Baltic sea sediments over thousands of years. ISME J. 7, 72–84 10.1038/ismej.2012.83 22832348 PMC3524260

[B24] DeFlaun M. F. Frederickson J. K. Dong H. Pfiffner S. M. Onstott T. C. Balkwill D. L. (2007). Isolation and characterization of a *Geobacillus thermoleovorans* strain from an ultra-deep South African gold mine. Syst. Appl. Microbiol. 30, 152–164 10.1016/j.syapm.2006.04.003 16709445

[B25] Detmers J. Schulte U. Strauss H. Kuever J. (2001). Sulfate reduction at a lignite seam: microbial abundance and activity. Microbial. Ecol. 42, 238–247 10.1007/s00248-001-1014-8 12024249

[B26] Detmers J. Strauss H. Schulte U. Bergmann A. Knittel K. Kuever J. (2004). Fish shows that *Desulfotomaculum* spp. are the dominating sulfate-reducing bacteria in a pristine aquifer. Microbial. Ecol. 47, 236–242 10.1007/s00248-004-9952-6 15085304

[B27] Felske A. Akkermans A. D. L. De Vos W. M. (1998). *In situ* detection of an uncultured predominant *Bacillus* in Dutch grassland soils. Appl. Environ. Microbiol. 64, 4588–4590 9797326 10.1128/aem.64.11.4588-4590.1998 PMC106688

[B28] Fichtel J. Köster J. Rullkötter J. Sass H. (2007). Spore dipicolinic acid contents used for estimating the number of endospores in sediments. FEMS Microbiol. Ecol. 61, 522–532 10.1111/j.1574-6941.2007.00354.x 17623026

[B29] Fichtel K. Mathes F. Könneke M. Cypionka H. Engelen B. (2012). Isolation of sulfate-reducing bacteria from sediments above the deep-subseafloor aquifer. Front. Microbiol. 3:65 10.3389/fmicb.2012.00065 22363336 PMC3282481

[B30] Filion G. Laflamme C. Turgeon N. Ho J. Duchaine C. (2009). Permeabilization and hybridization protocols for rapid detection of *Bacillus* spores using fluorescence *in situ* hybridization. J. Microbiol. Methods 77, 29–36 10.1016/j.mimet.2008.12.009 19159650

[B31] Finster K. (2008). Microbiological disproportionation of inorganic sulfur compounds. J. Sulfur Chem. 29, 281–292 10.1080/17415990802105770

[B32] Fischer K. Hahn D. Hönerlage W. Zeyer J. (1995). *In situ* detection of spores and vegetative cells of *Bacillus* megaterium in soil by whole cell hybridization. Syst. Appl. Microbiol. 18, 265–273 10.1016/S0723-2020(11)80397-8

[B33] Fliermans C. B. Balkwill D. L. (1989). Microbial life in deep terrestrial subsurfaces. Bioscience 39, 370–377 10.2307/1311066

[B34] Fredrickson J. K. Balkwill D. L. (2006). Geomicrobial processes and biodiversity in the deep terrestrial subsurface. Geomicrobiol. J. 23, 345–356 10.1080/01490450600875571

[B35] Gerasimchuk A. L. Shatalov A. A. Novikov A. L. Butorova O. P. Pimenov N. V. Lein A. Y. (2010). The search for sulfate-reducing bacteria in mat samples from the Lost City hydrothermal field by molecular cloning. Microbiology 79, 96–105 10.1134/S0026261710010133 20411667

[B36] Ghiorse W. C. Wilson J. T. (1988). Microbial ecology of the terrestrial subsurface. Adv. Appl. Microbiol. 33, 107–172 10.1016/S0065-2164(08)70206-5 3041739

[B37] Gieg L. M. Jack T. R. Foght J. M. (2011). Biological souring and mitigation in oil reservoirs. Appl. Microbiol. Biotechnol. 92, 263–282 10.1007/s00253-011-3542-6 21858492

[B38] Gittel A. Sorensen K. B. Skovhus T. L. Ingvorsen K. Schramm A. (2009). Prokaryotic community structure and sulfate reducer activity in water from high-temperature oil reservoirs with and without nitrate treatment. Appl. Environ. Microb. 75, 7086–7096 10.1128/AEM.01123-09 19801479 PMC2786513

[B39] Gold T. (1992). The deep, hot biosphere. Proc. Natl. Acad. Sci. U.S.A. 89, 6045–6049 10.1073/pnas.89.13.6045 1631089 PMC49434

[B40] Green B. D. Battisti L. Koehler T. M. Thorne C. B. Ivins B. E. (1985). Demonstration of a capsule plasmid in *Bacillus anthracis*. Infect. Immun. 49, 291–297 3926644 10.1128/iai.49.2.291-297.1985 PMC262013

[B41] Guan J. Xia L. P. Wang L. Y. Liu J. F. Gu J. D. Mu B. Z. (2013). Diversity and distribution of sulfate-reducing bacteria in four petroleum reservoirs detected by using 16s rRNA and *dsrAB* genes. Int. Biodeterior. Biodegr. 76, 58–66 10.1016/j.ibiod.2012.06.021

[B42] Hubert C. (2010). Microbial ecology of oil reservoir souring control by nitrate injection, in Handbook of Hydrocarbon and Lipid Microbiology, ed Timmis K. N (Berlin: Springer-Verlag), 2753–2766 10.1007/978-3-540-77587-4_204

[B43] Hubert C. Arnosti C. Brüchert V. Loy A. Vandieken V. Jørgensen B. B. (2010). Thermophilic anaerobes in Arctic marine sediments induced to mineralize complex organic matter at high temperature. Environ. Microbiol. 12, 1089–1104 10.1111/j.1462-2920.2010.02161.x 20192966

[B44] Hubert C. Loy A. Nickel M. Arnosti C. Baranyi C. Brüchert V. (2009). A constant flux of diverse thermophilic bacteria into the cold Arctic seabed. Science 325, 1541–1544 10.1126/science.1174012 19762643

[B45] Ingvorsen K. Zeikus J. G. Brock T. D. (1981). Dynamics of bacterial sulfate reduction in a eutrophic lake. Appl. Environ. Microb. 42, 1029–1036 16345898 10.1128/aem.42.6.1029-1036.1981 PMC244150

[B46] Isaksen M. F. Bak F. Jørgensen B. B. (1994). Thermophilic sulfate-reducing bacteria in cold marine sediment. FEMS Microbiol. Ecol. 14, 1–8 10.1111/j.1574-6941.1994.tb00084.x

[B47] Ishii K. Takii S. Fukunaga S. Aoki K. (2000). Characterization by denaturing gradient gel electrophoresis of bacterial communities in deep groundwater at the Kamaishi mine, Japan. J. Gen. Appl. Microbiol. 46, 85–93 10.2323/jgam.46.85 12483595

[B48] Itavaara M. Nyyssonen M. Kapanen A. Nousiainen A. Ahonen L. Kukkonen I. (2011). Characterization of bacterial diversity to a depth of 1500 m in the Outokumpu deep borehole, fennoscandian shield. FEMS Microbiol. Ecol. 77, 295–309 10.1111/j.1574-6941.2011.01111.x 21488910

[B49] Jackson B. E. Mcinerney M. J. (2000). Thiosulfate disproportionation by *Desulfotomaculum thermobenzoicum*. Appl. Environ. Microb. 66, 3650–3653 10.1128/AEM.66.8.3650-3653.2000 10919837 PMC92201

[B50] Johnson S. S. Hebsgaard M. B. Christensen T. R. Mastepanov M. Nielsen R. Munch K. (2007). Ancient bacteria show evidence of DNA repair. Proc. Natl. Acad. Sci. U.S.A. 104, 14401–14405 10.1073/pnas.0706787104 17728401 PMC1958816

[B51] Jones G. E. Starkey R. L. Feely H. W. Kulp J. L. (1956). Biological origin of native sulphur in salt domes of Texas and Louisiana. Science 123, 1124–1125 10.1126/science.123.3208.1124 17793426

[B52] Jørgensen B. B. (1977). The sulfur cycle of a coastal marine sediment (Limfjorden, Denmark). Limnol. Oceanogr. 22, 814–832 10.4319/lo.1977.22.5.0814

[B53] Jørgensen B. B. Boetius A. (2007). Feast and famine-microbial life in the deep-sea bed. Nat. Rev. Microbiol. 5, 770–781 10.1038/nrmicro1745 17828281

[B54] Jørgensen B. B. Nelson D. (2004). Sulfide oxidation in marine sediments: Geochemistry meets microbiology, in Sulfur Biogeochemistry - Past and Present, eds Amend J. P. Edwards K. J. Lyons T. W. (*Geol. Soc. Am*), 36–81 10.1130/0-8137-2379-5.63

[B55] Kakooei S. Ismail M. C. Ariwahjoedi B. (2012). Mechanisms of microbiology influenced corrosion: a review. World Appl. Sci. J. 17, 524–531

[B56] Kaksonen A. H. Spring S. Schumann P. Kroppenstedt R. M. Puhakka J. A. (2006). *Desulfotomaculum thermosubterraneum* sp. nov., a thermophilic sulfate-reducer isolated from an underground mine located in a geothermally active area. Int. J. Syst. Evol. Microbiol. 56, 2603–2608 10.1099/ijs.0.64439-0 17082399

[B57] Kaksonen A. H. Spring S. Schumann P. Kroppenstedt R. M. Puhakka J. A. (2007). *Desulfolvirgula thermocuniculi* gen. nov., sp. nov., a thermophilic sulfate-reducer isolated from a geothermal underground mine in Japan. Int. J. Syst. Evol. Microbiol. 57, 98–102 10.1099/ijs.0.64655-0 17220449

[B58] Karnachuk O. V. Pimenov N. V. Yusupov S. K. Frank Y. A. Puhakka Y. A. Ivanov M. V. (2006). Distribution, diversity, and activity of sulfate-reducing bacteria in the water column in Gek-Gel lake, Azerbaijan. Microbiology 75, 82–89 10.1134/S0026261706010152 16579451

[B59] Kieft T. L. Phelps T. J. Fredrickson J. K. (2007). Drilling, coring, and sampling subsurface environments, in Manual of Environmental Microbiology, 3rd Edn, eds Hurst C. J. Crawford R. L. Garland J. L. Lipson D. A. Mills A. L. Stezenbach L. D. (Washington, DC: ASM Press), 799–817

[B60] Klein M. Friedrich M. Roger A. J. Hugenholtz P. Fishbain S. Abicht H. (2001). Multiple lateral transfers of dissimilatory sulfite reductase genes between major lineages of sulfate-reducing prokaryotes. J. Bacteriol. 183, 6028–6035 10.1128/JB.183.20.6028-6035.2001 11567003 PMC99682

[B61] Klemps R. Cypionka H. Widdel F. Pfennig N. (1985). Growth with hydrogen, and further physiological-characteristics of *Desulfotomaculum* species. Arch. Microbiol. 143, 203–208 10.1007/BF00411048

[B62] Kotelnikova S. Pedersen K. (1997). Evidence for methanogenic *Archaea* and homoacetogenic *Bacteria* in deep granitic rock aquifers. FEMS Microbiol. Rev. 20, 339–349 10.1111/j.1574-6976.1997.tb00319.x

[B63] Kuever J. Rainey F. A. (2009). Desulfotomaculum, in Bergey's Manual of Systematic Bacteriology: The Firmicutes, eds Vos P. Garrity G. Jones D. Krieg N. R. Ludwig W. Rainey F. A. Schleifer K.-H. Whitman W. B. (Baltimore, MD: The Williams and Wilkins Co), 989–996

[B64] Kuever J. Rainey F. A. Hippe H. (1999). Description of *Desulfotomaculum* sp. groll as Desulfotomaculum gibsoniae sp. nov. Int. J. Syst. Bacteriol. 49, 1801–1808 10.1099/00207713-49-4-1801 10555363

[B65] Lan G. H. Li Z. T. Zhang H. Zou C. J. Qiao D. R. Cao Y. (2011). Enrichment and diversity analysis of the thermophilic microbes in a high temperature petroleum reservoir. Afr. J. Microbiol. Res. 5, 1850–1857 10.5897/AJMR11.354

[B66] Leloup J. Quillet L. Berthe T. Petit F. (2006). Diversity of the dsrAB (dissimilatory sulfite reductase) gene sequences retrieved from two contrasting mudflats of the Seine estuary, France. FEMS Microbiol. Ecol. 55, 230–238 10.1111/j.1574-6941.2005.00021.x 16420631

[B67] Leu J. Y. Mcgovern-Traa C. P. Porter A. J. R. Harris W. J. Hamilton W. A. (1998). Identification and phylogenetic analysis of thermophilic sulfate-reducing bacteria in oil field samples by 16s rRNA gene cloning and sequencing. Anaerobe 4, 165–174 10.1006/anae.1998.0156 16887637

[B68] Lever M. A. (2011). Acetogenesis in the energy-starved deep biosphere–a paradox. Front. Microbiol. 2:284 10.3389/fmicb.2011.00284 22347874 PMC3276360

[B69] Lever M. A. (2013). Functional gene surveys from ocean drilling expeditions - a review and perspective. FEMS Microbiol. Ecol. 84, 1–23 10.1111/1574-6941.12051 23228016

[B70] Lever M. A. Alperin M. Engelen B. Inagaki F. Nakagawa S. Steinsbu B. R. O. (2006). Trends in basalt and sediment core contamination during iodp expedition 301. Geomicrobiol. J. 23, 517–530 10.1080/01490450600897245

[B71] Lever M. A. Rouxel O. Alt J. C. Shimizu N. Ono S. Coggon R. M. (2013). Evidence for microbial carbon and sulfur cycling in deeply buried ridge flank basalt. Science 339, 1305–1308 10.1126/science.1229240 23493710

[B72] Lin L. H. Hall J. Lippmann−Pipke J. Ward J. A. Sherwood Lollar B. Deflaun M. (2005). Radiolytic H_2_ in continental crust: nuclear power for deep subsurface microbial communities. Geochem. Geophys. Geosyst. 6, Q07003 10.1029/2004GC000907

[B73] Lindahl T. (1993). Instability and decay of the primary structure of DNA. Nature 362, 709–715 10.1038/362709a0 8469282

[B74] Liu Y. J. Nikolausz M. Jin P. K. (2008). Abundance and diversity of sulphate-reducing bacteria within a crude oil gathering and transferring system in china. Ann. Microbiol. 58, 611–615 10.1007/BF0317556521072940

[B75] Liu Y. T. Karnauchow T. M. Jarrell K. F. Balkwill D. L. Drake G. R. Ringelberg D. (1997). Description of two new thermophilic *Desulfotomaculum* spp., *Desulfotomaculum putei* sp. nov, from a deep terrestrial subsurface, and Desulfotomaculum luciae sp. nov, from a hot spring. Int. J. Syst. Bacteriol. 47, 615–621 10.1099/00207713-47-3-615

[B76] Lomstein B. A. Jørgensen B. B. (2012). Pre-column liquid chromatographic determination of dipicolinic acid from bacterial endospores. Limnol. Oceanogr. Methods 10, 227–233 10.4319/lom.2012.10.227

[B77] Lomstein B. A. Langerhuus A. T. D'hondt S. Jørgensen B. B. Spivack A. J. (2012). Endospore abundance, microbial growth and necromass turnover in deep sub-seafloor sediment. Nature 484, 101–104 10.1038/nature10905 22425999

[B78] Love C. A. Patel B. K. C. Nichols P. D. Stackebrandt E. (1993). *Desulfotomaculum australicum*, sp. nov., a thermophilic sulfate-reducing bacterium isolated from the Great Artesian Basin of Australia. Syst. Appl. Microbiol. 16, 244–251 10.1016/S0723-2020(11)80475-3

[B79] Lovley D. R. Phillips E. J. (1994). Novel processes for anaerobic sulfate production from elemental sulfur by sulfate-reducing bacteria. Appl. Environ. Microbiol. 60, 2394–2399 16349323 10.1128/aem.60.7.2394-2399.1994 PMC201662

[B80] Luna V. A. King D. Davis C. Rycerz T. Ewert M. Cannons A. (2003). Novel sample preparation method for safe and rapid detection of *Bacillus anthracis* spores in environmental powders and nasal swabs. J. Clin. Microbiol. 41, 1252–1255 10.1128/JCM.41.3.1252-1255.2003 12624060 PMC150283

[B81] Magot M. (2005). Indigenous microbial communities in oil fields, in “Petroleum microbiology,” eds Ollivier B. Magot M. (Washington, DC: ASM), 21–34

[B82] Magot M. Ollivier B. Patel B. K. (2000). Microbiology of petroleum reservoirs. Antonie Van Leeuwenhoek 77, 103–116 10.1023/A:1002434330514 10768470

[B83] Ménez B. Pasini V. Brunelli D. (2012). Life in the hydrated suboceanic mantle. Nat. Geosci. 5, 133–137 10.1038/ngeo1359

[B84] Möller B. Oßmer R. Howard B. H. Gottschalk G. Hippe H. (1984). *Sporomusa*, a new genus of gram-negative anaerobic bacteria including *Sporomusa sphaeroides* sp. nov. and Sporomusa ovata sp. nov. Arch. Microbiol. 139, 388–396 10.1007/BF00408385

[B85] Morasch B. Schink B. Tebbe C. C. Meckenstock R. U. (2004). Degradation of o-xylene and m-xylene by a novel sulfate-reducer belonging to the genus *Desulfotomaculum*. Arch. Microbiol. 181, 407–417 10.1007/s00203-004-0672-6 15127183

[B86] Morita R. Zobell C. (1955). Occurrence of bacteria in pelagic sediments collected during the Mid-Pacific Expedition. Deep-Sea Res. 3, 66–73 10.1016/0146-6313(55)90036-8

[B87] Moser D. P. Gihring T. M. Brockman F. J. Fredrickson J. K. Balkwill D. L. Dollhopf M. E. (2005). *Desulfotomaculum* and *Methanobacterium* spp. Dominate a 4- to 5-kilometer-deep fault. Appl. Environ. Microbiol. 71, 8773–8783 10.1128/AEM.71.12.8773-8783.2005 16332873 PMC1317344

[B88] Nakagawa T. Hanada S. Maruyama A. Marumo K. Urabe T. Fukui M. (2002). Distribution and diversity of thermophilic sulfate-reducing bacteria within a cu-pb-zn mine (Toyoha, Japan). FEMS Microbiol. Ecol. 41, 199–209 10.1111/j.1574-6941.2002.tb00981.x 19709254

[B89] Nazina T. Rozanova E. (1978). Thermophillic sulfate-reducing bacteria from oil-bearing strata. Mikrobiologiia 47, 142–148 651683

[B90] Nazina T. N. Ivanova A. E. Kanchaveli L. P. Rozanova E. P. (1989). A new spore-forming thermophilic methylotrophic sulfate-reducing bacterium, *Desulfotomaculum kuznetsovii* sp. nov. Microbiology 57, 659–663

[B91] Nazina T. N. Rozanova E. P. Belyakova E. V. Lysenko A. M. Poltaraus A. B. Tourova T. P. (2005). Description of “*Desulfotomaculum nigrificans* subsp. salinus” as a new species, Desulfotomaculum salinum sp. nov. Microbiology 74, 567–574 10.1007/s11021-005-0104-x 16315984

[B92] Nealson K. H. Ford J. (1980). Surface enhancement of bacterial manganese oxidation: Implications for aquatic environments. Geomicrobiol. J. 2, 21–37 10.1080/01490458009377748

[B93] Nilsen R. K. Beeder J. Thorstenson T. Torsvik T. (1996a). Distribution of thermophilic marine sulfate reducers in North sea oil field waters and oil reservoirs. Appl. Environ. Microbiol. 62, 1793–1798 16535321 10.1128/aem.62.5.1793-1798.1996 PMC1388859

[B94] Nilsen R. K. Torsvik T. Lien T. (1996b). *Desulfotomaculum thermocisternum* sp. nov, a sulfate reducer isolated from a hot North sea oil reservoir. Int. J. Syst. Bacteriol. 46, 397–402 10.1099/00207713-46-2-397

[B95] Ogg C. D. Patel B. K. (2011). *Desulfotomaculum varum* sp. nov., a moderately thermophilic sulfate-reducing bacterium isolated from a microbial mat colonizing a great artesian basin bore well runoff channel. 3 Biotech. 1, 139–149 10.1007/s13205-011-0017-5 22611525 PMC3339622

[B96] Ollivier B. Alazard D. (2010). The oil reservoir ecosystem, in Handbook of Hydrocarbon and Lipid Microbiology, ed Timmis K. N. (Berlin: Springer-Verlag), 2262–2268 10.1007/978-3-540-77587-4_164

[B97] Ollivier B. Cayol J.-L. Fauque G. (2007). Sulphate-reducing bacteria from oil fields environments and deep-sea hydrothermal vents, in Sulphate-Reducing Bacteria: Environmental and Engineered Systems, eds Barton L. Hamilton W. (London: Cambridge University Press), 305–328 10.1017/CBO9780511541490.011

[B99] Ollivier B. Magot M. (2005). Petroleum Microbiology. Washington, DC: ASM Press

[B100] Oren A. (2012). There must be an acetogen somewhere. Front. Microbiol. 3:22 10.3389/fmicb.2012.00022 22319520 PMC3269027

[B101] Orsi W. D. Edgcomb V. P. Christman G. D. Biddle J. F. (2013). Gene expression in the deep biosphere. Nature 499, 205–208 10.1038/nature12230 23760485

[B102] Parkes R. J. Cragg B. A. Wellsbury P. (2000). Recent studies on bacterial populations and processes in subseafloor sediments: a review. Hydrogeol. J. 8, 11–28 10.1007/PL00010971

[B103] Parshina S. N. Sipma J. Nakashimada Y. Henstra A. M. Smidt H. Lysenko A. M. (2005). *Desulfotomaculum carboxydivorans* sp. nov., a novel sulfate-reducing bacterium capable of growth at 100% CO. Int. J. Syst. Evol. Microbiol. 55, 2159–2165 10.1099/ijs.0.63780-0 16166725

[B104] Pedersen K. (1993). The deep subterranean biosphere. Earth-Sci. Rev. 34, 243–260 10.1016/0012-8252(93)90058-F

[B105] Pedersen K. (1997). Microbial life in deep granitic rock. FEMS Microbiol. Rev. 20, 399–414 10.1111/j.1574-6976.1997.tb00325.x11540481

[B106] Pedersen K. (2000). Exploration of deep intraterrestrial microbial life: current perspectives. FEMS Microbiol. Lett. 185, 9–16 10.1111/j.1574-6968.2000.tb09033.x 10731600

[B107] Philippot P. Van Zuilen M. Lepot K. Thomazo C. Farquhar J. Van Kranendonk M. J. (2007). Early archaean microorganisms preferred elemental sulfur, not sulfate. Science 317, 1534–1537 10.1126/science.1145861 17872441

[B109] Reed D. W. Fujita Y. Delwiche M. E. Blackwelder D. B. Sheridan P. P. Uchida T. (2002). Microbial communities from methane hydrate-bearing deep marine sediments in a forearc basin. Appl. Environ. Microbiol. 68, 3759–3770 10.1128/AEM.68.8.3759-3770.2002 12147470 PMC124055

[B110] Reith F. (2011). Life in the deep subsurface. Geology 39, 287–288 10.1130/focus032011.1

[B111] Riedinger N. Brunner B. Formolo M. Solomon E. Kasten S. Strasser M. (2010). Oxidative sulfur cycling in the deep biosphere of the Nankai trough, Japan. Geology 38, 851–854 10.1130/G31085.1

[B112] Rosnes J. T. Torsvik T. Lien T. (1991). Spore-forming thermophilic sulfate-reducing bacteria isolated from North sea-oil field waters. Appl. Environ. Microbiol. 57, 2302–2307 16348538 10.1128/aem.57.8.2302-2307.1991 PMC183567

[B113] Russell B. F. Phelps T. J. Griffin W. T. Sargent K. A. (1992). Procedures for sampling deep subsurface microbial communities in unconsolidated sediments. Ground Water Monit. Remediation 12, 96–104 10.1111/j.1745-6592.1992.tb00414.x

[B114] Santelli C. M. Banerjee N. Bach W. Edwards K. J. (2010). Tapping the subsurface ocean crust biosphere: low biomass and drilling-related contamination calls for improved quality controls. Geomicrobiol. J. 27, 158–169 10.1080/01490450903456780

[B115] Sass H. Cypionka H. Babenzien H.-D. (1997). Vertical distribution of sulfate-reducing bacteria at the oxic-anoxic interface in sediments of the oligotrophic lake Stechlin. FEMS Microbiol. Ecol. 22, 245–255 10.1111/j.1574-6941.1997.tb00377.x

[B116] Sass H. Wieringa E. Cypionka H. Babenzien H. D. Overmann J. (1998). High genetic and physiological diversity of sulfate-reducing bacteria isolated from an oligotrophic lake sediment. Arch. Microbiol. 170, 243–251 10.1007/s002030050639 9732438

[B117] Schrenk M. O. Brazelton W. J. Lang S. Q. (2013). Serpentinization, carbon, and deep life. Rev. Mineral. Geochem. 75, 575–606 10.2138/rmg.2013.75.18

[B118] Sinclair J. Ghiorse W. (1989). Distribution of aerobic bacteria, *Protozoa*, *Algae*, and *Fungi* in deep subsurface sediments. Geomicrobiol. J. 7, 15–31 10.1080/01490458909377847

[B119] Slobodkin A. I. Reysenbach A.-L. Slobodkina G. B. Baslerov R. V. Kostrikina N. A. Wagner I. D. (2012). *Thermosulfurimonas dismutans* gen. nov., sp. nov., an extremely thermophilic sulfur-disproportionating bacterium from a deep-sea hydrothermal vent. Int. J. Syst. Evol. Microbiol. 62, 2565–2571 10.1099/ijs.0.034397-0 22199218

[B120] Smith D. C. Spivack A. J. Fisk M. R. Haveman S. A. Staudigel H. Party L. (2000). Methods for quantifying potential microbial contamination during deep ocean coring. Ocean Drill. Program Tech. Note 28 10.2973/odp.tn.28.2000

[B121] Stahl D. A. Fishbain S. Klein M. Baker B. J. Wagner M. (2002). Origins and diversification of sulfate-respiring microorganisms. Anton. Leeuw. Int. J. G. 81, 189–195 10.1023/A:1020506415921 12448717

[B122] Stetter K. Huber R. Blöchl E. Kurr M. Eden R. Fielder M. (1993). Hyperthermophilic archaea are thriving in deep north sea and alaskan oil reservoirs. Nature 365, 743–745 10.1038/365743a0

[B123] Stevens T. O. Mckinley J. P. (1995). Lithoautotrophic microbial ecosystems in deep basalt aquifers. Science 270, 450–455 10.1126/science.270.5235.450

[B124] Szewzyk U. Szewzyk R. Stenström T. A. (1994). Thermophilic, anaerobic bacteria isolated fromm a deep borehole in granite in Sweden. Proc. Natl. Acad. Sci. U.S.A. 91, 1810–1813 10.1073/pnas.91.5.1810 11607462 PMC43253

[B125] Tardy-Jacquenod C. Caumette P. Matheron R. Lanau C. Arnauld O. Magot M. (1996). Characterization of sulfate-reducing bacteria isolated from oil-field waters. Can. J. Microbiol. 42, 259–266 10.1139/m96-038 8868233

[B126] Tardy-Jacquenod C. Magot M. Patel B. K. C. Matheron R. Caumette P. (1998). *Desulfotomaculum halophilum* sp. nov., a halophilic sulfate-reducing bacterium isolated from oil production facilities. Int. J. Syst. Bacteriol. 48, 333–338 10.1099/00207713-48-2-333 9731271

[B127] Tasaki M. Kamagata Y. Nakamura K. Mikami E. (1991). Isolation and characterization of a thermophilic benzoate-degrading, sulfate-reducing bacterium, *Desulfotomaculum thermobenzoicum* sp. *nov*. Arch. Microbiol. 155, 348–352 10.1007/BF00243454

[B128] Tebo B. M. Obraztsova A. Y. (1998). Sulfate-reducing bacterium grows with cr(vi), u(vi), mn(iv), and fe(iii) as electron acceptors. FEMS Microbiol. Lett. 162, 193–198 10.1111/j.1574-6968.1998.tb12998.x

[B129] Teske A. P. (2006). Microbial communities of deep marine subsurface sediments: Molecular and cultivation surveys. Geomicrobiol. J. 23, 357–368 10.1080/01490450600875613

[B130] Thamdrup B. Finster K. Hansen J. W. Bak F. (1993). Bacterial disproportionation of elemental sulfur coupled to chemical reduction of iron or manganese. Appl. Environ. Microbiol. 59, 101–108 16348835 10.1128/aem.59.1.101-108.1993 PMC202062

[B131] Thomas M. C. Shields M. J. Hahn K. R. Janzen T. W. Goji N. Amoako K. K. (2013). Evaluation of DNA extraction methods for *Bacillus anthracis* spores isolated from spiked food samples. J. Appl. Microbiol. 115, 156–162 10.1111/jam.12206 23560745

[B132] Tritla J. Alonso-Azcárate J. Bottrell S. H. (2000). Molten sulphur-dominated fluids in the origin of a native sulphur mineralization in lacustrine evaporites from Cervera del Rio Alhama (Cameros Basin, NE Spain). J. Geochem. Explor. 69–70, 183–187. 10.1016/S0375-6742(00)00023-6

[B133] Vandieken V. Knoblauch C. Jørgensen B. B. (2006). *Desulfotomaculum arcticum* sp. nov., a novel spore-forming, moderately thermophilic, sulfate-reducing bacterium isolated from a permanently cold fjord sediment of Svalbard. Int. J. Syst. Evol. Microbiol. 56, 687–690 10.1099/ijs.0.64058-0 16585677

[B134] Vreeland R. H. Rosenzweig W. D. Powers D. W. (2000). Isolation of a 250 million-year-old halotolerant bacterium from a primary salt crystal. Nature 407, 897–900 10.1038/35038060 11057666

[B135] Wang P. Xiao X. Zhang H. Wang F. (2008). Molecular survey of sulphate-reducing bacteria in the deep-sea sediments of the West Pacific warm pool. J. Ocean. Univ. Chin. 7, 269–275 10.1007/s11802-008-0269-9

[B136] Weerasekara M. L. Ryuda N. Miyamoto H. Okumura T. Ueno D. Inoue K. (2013). Double-color fluorescence *in situ* hybridization (FISH) for the detection of *Bacillus anthracis* spores in environmental samples with a novel permeabilization protocol. J. Microbiol. Methods 93, 177–184 10.1016/j.mimet.2013.03.007 23523967

[B137] Whitman W. B. Coleman D. C. Wiebe W. J. (1998). Prokaryotes: the unseen majority. Proc. Natl. Acad. Sci. U.S.A. 95, 6578–6583 10.1073/pnas.95.12.6578 9618454 PMC33863

[B138] Widdel F. (1988). Microbiology and ecology of sulfate and sulfur-degrading bacteria, in Biology of Anaerobic Microorganisms, ed Zehnder A. J. B. (New York, NY: Wiley-Interscience), 469–585

[B139] Zhuang W. Q. Tay J. H. Maszenan A. Krumholz L. Tay S. L. (2003). Importance of gram−positive naphthalene−degrading bacteria in oil−contaminated tropical marine sediments. Lett. Appl. Microbiol. 36, 251–257 10.1046/j.1472-765X.2003.01297.x 12641721

[B140] Ziegenbalg S. B. Brunner B. Rouchy J. M. Birgel D. Pierre C. Böttcher M. E. (2010). Formation of secondary carbonates and native sulphur in sulphate-rich Messinian strata, Sicily. Sediment. Geol. 227, 37–50 10.1016/j.sedgeo.2010.03.007

